# mTOR inhibition and levels of the DNA repair protein MGMT in T98G glioblastoma cells

**DOI:** 10.1186/1476-4598-13-144

**Published:** 2014-06-08

**Authors:** Sarah Smalley, Anthony J Chalmers, Simon J Morley

**Affiliations:** 1 Department of Biochemistry, School of Life Sciences, University of Sussex, Brighton BN1 9QG, UK; 2 University of Glasgow, Beatson Institute for Cancer Research and Beatson West of Scotland Cancer Centre, 1053 Great Western Road, Glasgow G12 0YN, UK

**Keywords:** MGMT, Stability, Translation, mTOR, Initiation of translation, KU0063794, Glioblastoma, TMZ resistance

## Abstract

**Background:**

Glioblastoma multiforme (GBM), the most common and most aggressive type of primary adult brain tumour, responds poorly to conventional treatment. Temozolomide (TMZ) chemotherapy remains the most commonly used treatment, despite a large proportion of tumours displaying TMZ resistance. 60% of GBM tumours have unmethylated *MGMT* promoter regions, resulting in an overexpression of the DNA repair protein O^6^-methylguanine-DNA methyltransferase (MGMT), which is responsible for tumour resistance to TMZ chemotherapy. Tumours also often exhibit hyperactive PI3-kinase/mTOR signalling, which enables them to resynthesise proteins quickly. Since MGMT is a suicide protein that is degraded upon binding to and repairing TMZ-induced O^6^-methylguanine adducts, it has been hypothesized that inhibition of translation via the mTOR signalling pathway could generate a tumour-specific reduction in MGMT protein and increase TMZ sensitivity.

**Methods:**

MGMT was monitored at the post-transcriptional, translational and protein levels, to determine what effect mTOR inhibition was having on MGMT protein expression *in vitro*.

**Results:**

We show that inhibiting mTOR signalling is indeed associated with acute inhibition of protein synthesis. Western blots show that despite this, relative to loading control proteins, steady state levels of MGMT protein *increased* and MGMT mRNA was retained in heavy polysomes. Whilst TMZ treatment resulted in maintained MGMT protein levels, concomitant treatment of T98G cells with TMZ and KU0063794 resulted in increased MGMT protein levels without changes in total mRNA levels.

**Conclusions:**

These *in vitro* data suggest that, counterintuitively, mTOR inhibition may not be a useful adjunct to TMZ therapy and that more investigation is needed before applying mTOR inhibitors in a clinical setting.

## Introduction

Glioblastoma (GBM), a WHO grade IV astrocytoma, is the most common [1] and most aggressive type of primary brain tumour. Current treatment consists of tumour resection (where possible), followed by ionising radiotherapy combined with concomitant and adjuvant temozolomide (TMZ) chemotherapy [2]. TMZ is a methylating agent that creates lesions in DNA, the most cytotoxic of which is O^6^-methylguanine [3]. This multimodal treatment regimen is rigorous, yet prognosis remains poor and although TMZ chemotherapy improves survival in a subset of patients, 75% die within 2 years and the vast majority of patients experience disease recurrence [4].

O^6^-methylguanine-DNA methyltransferase (MGMT) is a DNA repair protein, which removes O^6^-methylguanine adducts from damaged DNA [5]. This reaction is irreversible and once bound to the alkyl group MGMT is ubiquitinated and destroyed by the proteasome [6]. The cytotoxic effect of TMZ chemotherapy is therefore influenced by the ability of tumour cells to re-synthesise MGMT and maintain steady state levels of the protein. Previous work has shown that *MGMT* promoter methylation resulting in gene silencing and resultant low levels of MGMT protein, increases sensitivity to TMZ and is associated with improved patient survival. Unfortunately direct inhibition of MGMT using small molecule inhibitors such as lomeguatrib has not been successful as a clinical application because it also increases haematological toxicity [2,7,8]. However, downregulation of MGMT remains an attractive therapeutic strategy for patients with tumours exhibiting unmethylated *MGMT* promoters if it could be achieved in a tumour specific manner.

Common mutations in GBM cells include genetic changes that result in a loss of PTEN function and EGFR amplification [9], both of which can generate hyperactive phosphoinositide 3-kinase (PI3K)/mTOR signalling. mTOR is a serine/threonine kinase that belongs to the PI3K-related kinase family and interacts with proteins to form two distinct complexes in mammals, mTORC1 and mTORC2 [10]. mTORC1, when active, regulates protein translation through the phosphorylation of 4EBP1. Phosphorylation of 4E-BP1 prevents it binding to eIF4E, which enables eIF4E to participate in the formation of the eIF4F complex on the m^7^ GTP cap structure of mRNA and mediate small ribosomal subunit binding and subsequent protein translation [11]. The hyperactivity of this pathway therefore results in increased protein synthesis, promoting cell growth and proliferation.

Because of this there has been interest in the use of mTOR inhibitors in combination with radiation and TMZ in the treatment of GBM. The rationale for combining mTOR inhibitors with TMZ treatment is based on the reasoning that the lesions in DNA caused by TMZ will result in a depletion of cellular MGMT protein levels. When coupled with mTOR inhibition, not only would there be a decrease in MGMT levels, but the tumour cell would be compromised in its ability to synthesise new protein, thus sensitising the cells to further TMZ treatment. In addition to this, tumour cells should be specifically targeted with this course of treatment, due to the tumour cells oncogenic addiction to the PI3K/mTOR signalling pathway. This would avoid the current drawbacks faced during direct inhibition of MGMT, as it would avoid MGMT depletion in healthy cells, and therefore avoid undesirable cytotoxicity.

In this work, we have used Western blotting to examine the effect of inhibiting mTORC1/2 signalling on steady state MGMT protein levels in T98G GBM cells, a cell line which exhibits relatively high MGMT expression compared to other glioblastoma cell lines [12,13]. KU0063794 is a specific mTORC1 and mTORC2 inhibitor that does not display significant activity against similar kinases such as PI3K, ERK1/2, or p38MAPK [14].

The findings described in this paper are of both biochemical and potential clinical interest. The research highlights how important it is to identify how DNA repair proteins are translated and maintained as proteins, which is an important consideration, especially when manipulating them for clinical benefit.

## Materials and methods

### Cell culture

T98G (ECACC) cells were cultured to confluency in minimum essential medium (MEM) supplemented with 5% non-essential amino acids (Invitrogen, UK), 10% foetal calf serum (Biosera, UK) and 5% GlutaMax (Invitrogen, UK). U87 (ECACC) cells were cultured in the same manner but with Hyclone foetal calf serum.

### Concentrations of drug treatments

Cells were treated with the following concentrations of drugs: 10 μM KU0063794, 10 μM TMZ and 10 μM emetine.

### Preparation of cell extracts

Following treatment, cells were isolated in a cooled microfuge and washed briefly with 0.5 ml ice-cold PBS. Pellets were resuspended in 100 μl ice-cold lysis buffer (20 mM MOPS-KOH, pH 7.2, 20 mM sodium fluoride, 1 μM microcystin LR, 75 mM KCl, 2 mM MgCl_2_, 2 mM benzamidine, 2 mM Na_3_VO_4_, complete protease inhibitor mix (−EDTA (Roche, UK), 0.1% (v/v) SDS), with the addition of 0.5% (v/v) Igepal and 0.5% (w/v) deoxycholate (DOC) with vortexing. Cell debris was removed by centrifugation in a microfuge for 5 min at 4°C and the resultant supernatants recovered.

### Western blotting

Samples containing equal amounts of protein (10 μg) were resolved by polyacrylamide gel electrophoresis (SDS-PAGE) and processed as described previously [15,16]. Briefly, membranes were blocked using TBS-Tween containing 3% (w/v) BSA for 1 hour and incubated with antisera diluted in the same overnight at 4°C. Following washing in TBS-Tween, membranes were incubated with horseradish peroxidase-conjugated secondary antibody and signals developed using ECL reagent. The antiserum used were raised against: eIF4A, phospho-eIF4E, eIF4G, eIF4E [15,16]; MGMT, phospho-eIF2α, (Abcam, UK); 4E-BP1, phospho-4E-BP S65, phospho-4E-BP1 T70, phospho-p70 S6K T389, phospho- ERK1/2, phospho-p38 MAPK, phospho-rpS6 S240/44, phospho-Akt T308, p21^cip1^ (Cell Signaling, UK).

### [^35^S]-methionine labelling of cellular protein

One hour prior to harvesting, T98G cells were pulse-labelled with [^35^S]-methionine (MP Biomedicals, UK; 10 μCi/ml) and cell extracts prepared as above and 5 μl of extract was spotted onto Whatman filter papers. Filters were transferred to 10% (v/v) trichloroacetic acid (TCA) containing 5 mM unlabelled methionine for 15 minutes then boiled in 5% (v/v) TCA. Once cooled, the filters were washed in ethanol, then acetone, dried and subjected to liquid scintillation counting. Protein synthesis, expressed as cpm/ μg protein, is shown as a % of the rate obtained in cells incubated in the absence of drugs.

### MTS assay

Cells were cultured in a 96 well plate in 100 μl of complete medium. Following incubation with drugs, 20 μl of MTS/PMS solution (Promega, UK) was added, and left for 2 hours at 37°C. The colour reaction, reflecting metabolic activity was quantified by measuring absorbance at A^490^ using the control wells for comparison. All assays were carried out in triplicate.

### Flow cytommetry

Following incubation of cells as described in the figure legends, cells were washed with warm PBS then trypsinised and removed from culture dishes. Cells were washed in PBS and fixed in 70% ethanol/30% PBS at 4°C overnight. Immediately before analysis, the cells were washed in PBS and re-suspended in 500 μl PBS containing 0.1 mg/ml RNase A (Sigma, UK) and 30 μg/ml propidium iodide (Invitrogen, UK) and analysed by a FACSCanto™ flow cytometer (BD Biosciences) using BD FACSDiva™ software. Data shown are representative of those obtained in at least three separate experiments.

### Quantitative RT-PCR

RNA was extracted from cell extracts using an RNA easy mini-kit (Qiagen, UK) as per manufacturer’s instructions. RNA concentration was then quantified using a Nanodrop and 1 μg of RNA was used for cDNA synthesis using the Promega ImpromII kit. The SYBR real-time PCR system (Kapa Biosystems, UK) was used to quantify transcript abundance for genes of interest and18S mRNA was used as a control. Template equivalent to 5 ng of RNA in cDNA library per reaction was added to each 20 μl reaction with a final primer concentration of 200 nM per reaction. Crossing thresholds were determined using MxPro software (Agilent), and fold-difference in RNA quantity was calculated using the relative quantification method (2^-ΔΔct^).

### Immunofluorescence

Cells were plated on glass coverslips 8 hours before treatment. Cells were then fixed with 4% (w/v) paraformaldehyde for 20 minutes at room temperature and permeabilised for 5 minutes in 0.1% Triton X-100/PBS. Cells were blocked for 20 minutes with 5% (w/v) gelatin/PBS in a humidified chamber. Cells were incubated with anti-MGMT (Abcam, UK) for 1 hour at room temperature also in a humidified chamber. Alexa Fluor 555-conjugated anti-rabbit secondary antibody was added for 1 hour at room temperature. Alexa Fluor 488 phalloidin was also used in the secondary antibody incubation. Following further extensive washing, nuclei were stained with DAPI (4’, 6-diamidino-2-phenylindole) for 5 minutes. After a further two washes, coverslips were mounted on microscope slides with Mowiol mounting solution (0.2 M Tris/HCl (pH 8.5), 33% (w/v) glycerol, 13% (w/v) Mowiol and 2.5% (w/v) DABCO (1, 4-diazobicyol [2]-octane)) and sealed with clear nail polish. Images were collected on a Zeiss Axiovert LSM510 scanning confocal microscope using a × 100 objective. Single stain, bleed-though controls and antibody cross-reaction controls were prepared for each sample (results not shown).

### m^7^GTP-Sepharose affinity chromatography

To isolate eIF4E and associated proteins [15,16] cells were lysed as described and aliquots containing 100 μg were incubated with 30 μl (50% v/v in 1 mg/ml cytochrome c) m^7^GTP-Sepharose resin for 15 mins on ice, with mixing. The resin was washed twice in 200 μl buffer (20 mM MOPS/KOH pH 7.2, 75 mM KCl, 2 mM benzamidine, 7 mM 2-mercaptoethanol, 2 mM MgCl_2_, 0.1 mM GTP, complete protease inhibitor mix (−EDTA), 25 mM NaF). To visualise eIF4E and associated proteins, the resin was washed twice with 200 μl buffer then eluted in SDS-PAGE sample buffer containing 10% w/v β-mercaptoethanol prior to Western blotting.

### Polysome profiles

Cells were treated with 100 μg/ml of cycloheximide (Sigma, UK) for 3 mins before harvesting. The cells were washed twice in PBS containing 100 μg/ml cycloheximide and lysed in hypertonic lysis buffer (10 mM Tris/HCl pH7.5, 10 mM KCl, 15 mM MgCl_2_, 1%(v/v) Igepal, 0.5%(v/v) DOC, 40 mM β-glycerophosphate, complete protease inhibitor mix (−EDTA), 2 mM DTT, 100 μg/ml cycloheximide, 10 U/ml RNase inhibitor) for 15 minutes on ice and homogenised every 5 mins. The cell lysate was centrifuged at 14,000 rpm for 3 mins and the supernatant was separated on a 11.2 ml sucrose gradient (10-60% (w/v) in hypertonic lysis buffer) for 130 mins at 38, 000 rpm using a Beckman SW40 rotor and fractionated using an ISCO, monitoring A^260^ nm through a flow cell. For analysis of GAPDH and MGMT mRNA, the mRNA from pooled fractions was extracted and quantitative RT-PCR was used to determine mRNA distribution across each fraction.

## Results

### TMZ does not affect steady state levels of MGMT protein or protein synthesis in T98G cells

**Figure 1:**
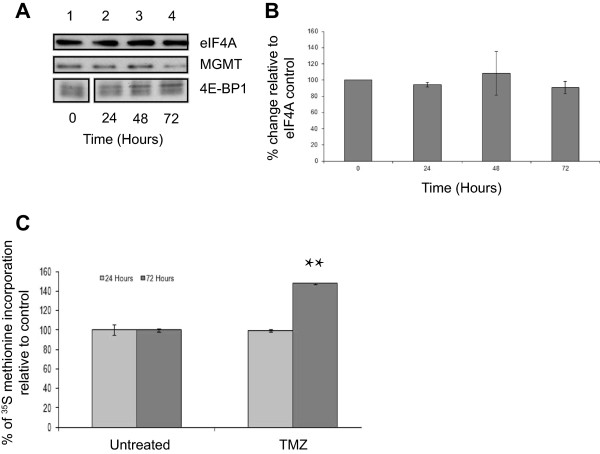
**TMZ treatment does not affect steady state levels of MGMT protein.** T98G cells were incubated in the absence (lane 1) or presence of TMZ for 24 hours (lane 2), 48 hours (lanes 3) or 72 hours (lane 4). **A**. Proteins were visualised by Western blotting using the antiserum indicated. **B**. MGMT protein levels in **(A)** were quantified and expressed relative to the eIF4A loading control. Error bars are the SE (n = 3). Confidence limits were set: *p = <0.2, **p = <0.05 ***p = <0.005. **C**. Cells were incubated with [^35^S] methionine, as described in Materials and Methods. Incorporation of radioactive methionine into protein was determined as cpm/μg protein; results are presented as a % of methionine incorporated in to cells incubated in the absence of TMZ. Error bars are the S.D (n = 3).

TMZ treatment causes damage to DNA, creating O^6^-methylguanine lesions, which are repaired by MGMT [17,18]. As TMZ is the standard treatment for patients with GBM [2], we investigated its effect on mTORC1 signalling and MGMT levels in the T98G GBM cell line. Cells were incubated with TMZ over a 72 hour time period. This time point was chosen as treatment regimens are routinely administered between 1 and 5 days each month to patients, due to a low tolerance of the drug [4,19]. SDS-PAGE and Western blotting revealed that, when expressed relative to eIF4A levels used as a protein loading control, TMZ does not result in a significant change in MGMT protein levels between 24 and 72 hours of incubation (Figure 1A). Quantification of these data from three separate experiments (using eIF4A as a loading control; error bars are SE) showed that although there was some experimental variability between time-courses, there was no significant difference in MGMT protein levels following treatment of cells with TMZ (Figure 1B). Similar results were obtained when alpha tubulin was used as a loading control to normalise levels of MGMT protein between experiments (see below). Neither FACS analysis (Additional file 1: Figure S1) nor mitochondrial toxicity assays (Additional file 1: Figure S2) showed any effect of TMZ on cell death under these assay conditions. 4E-BP1 remained hyperphosphorylated in the presence of TMZ indicating that mTORC1 signalling was largely unaffected under these conditions, even at 72 hours (Additional file 1: Figure S3). Protein synthesis assays (Figure 1C; error bars are SE; n = 3) showed that TMZ did not inhibit protein synthesis rates relative to the untreated cells at 24 hours; indeed rates appeared to be increased at 72 hours (p = 0.012).

### Concomitant TMZ treatment with KU0063794 causes an increase in MGMT protein levels

**Figure 2:**
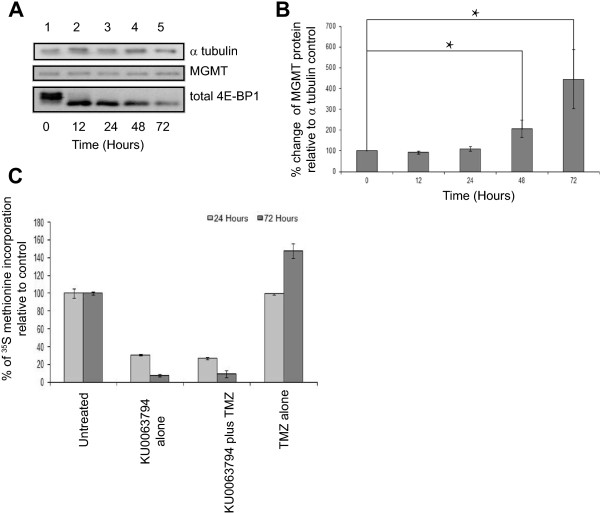
**TMZ combined with KU0063794 decreases MGMT protein levels compared to treatments with KU0063794 alone**, **but an increase in overall MGMT protein levels is still observed.** T98G cells were incubated in the absence (lane 1) or presence of TMZ and KU0063794 for 12 hours (lane 2), 24 hours (lanes 3), 48 hours (lane 4) or 72 hours (lane 5). **A** Proteins were visualised by Western blotting using the antiserum indicated. **B**. MGMT protein levels in **(A)** were quantified and expressed relative to the α tubulin loading control. Error bars are the SE (n = 3). Confidence limits were set: *p = <0.2, **p = <0.05 ***p = <0.005. **C**. Cells were incubated in the absence or presence of KU0063794, TMZ and KU0063794 or TMZ alone. Cells were incubated with [^35^S] methionine, as described in Materials and Methods. Incorporation of radioactive methionine into protein was determined as cpm/μg protein; results are presented as a% of methionine incorporated in to cells incubated in the absence of any treatments. Error bars are the S.D (n = 3).

To determine if combining mTOR inhibition with TMZ chemotherapy effectively reduced MGMT protein levels, we combined KU0063794 with TMZ and monitored 4E-BP1 phosphorylation and MGMT protein levels by SDS-PAGE and Western blotting, using alpha tubulin as a protein loading control (Figure 2A). 4E-BP1 is dephosphorylated in cells treated with both KU0063794 and TMZ, which is characteristic of mTORC1 inhibition. This is consistent with the finding that concomitant treatment of cells with both TMZ and KU0063794 resulted in the inhibition of p70S6K and increased phosphorylation of Akt Thr308 (Additional file 1: Figure S4), both of which indicate mTORC1 inhibition. Under these conditions, surprisingly, when protein loading is taken into consideration using alpha tubulin, levels of MGMT protein increased (Figure 2A). Quantification of these data from three separate experiments (using alpha tubulin as a loading control; error bars are SE) showed that although there was experimental variability between time-courses, there was a statistically significant change in protein levels at 48 and 72 hours (p = 0.12 and 0.13, respectively) (Figure 2B). These data suggest that increase in MGMT levels is most likely brought about by KU0063794 and overprinted the lack of MGMT protein level change seen in the TMZ treated cells alone (Figure 1). This was in spite of the fact that protein synthesis rates were inhibited to the same level with KU0063794 whether TMZ was present or not (Figure 2C). Confocal microscopy was used to determine the localisation of the MGMT protein within the cell (Additional file 1: Figure S5), but, MGMT was not differentially localised upon drug treatments, indicating that the change in MGMT protein levels was not a reflection of MGMT transport between cellular compartments.

### KU0063794 treatment is responsible for the dramatic rise in MGMT protein levels although global translation remains reduced

**Figure 3:**
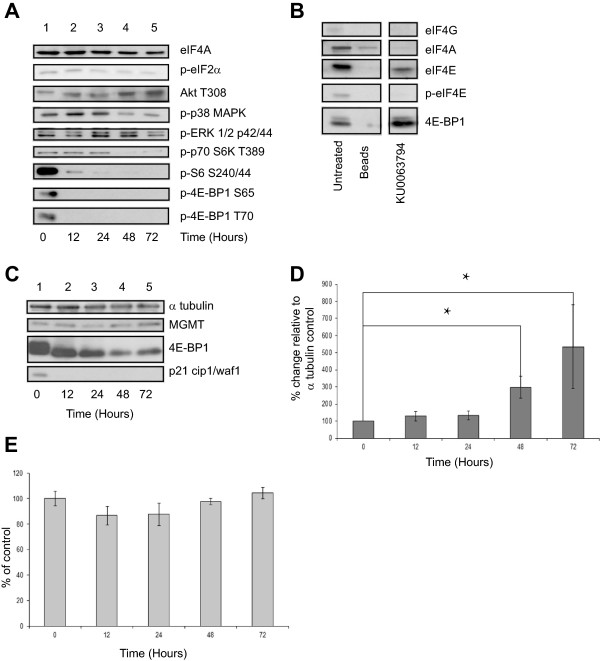
**KU0063794 inhibits protein translation via mTOR kinase inhibition in T98G cells but MGMT protein levels are increased.** T98G cells were incubated in the absence (lane 1) or presence of KU0063794 for 12 hours (lane 2), 24 hours (lanes 3), 48 hours (lane 4) or 72 hours (lane 5). **A**. Western blotting was carried out as described in Materials and Methods **B**. Aliquots of protein extract were subjected to m^7^GTP-Sepharose affinity chromatography as described in Materials and Methods. Western blotting was carried out. All lanes were resolved on the same gel. **C**. T98G cells were incubated in the absence (lane 1) or presence of KU0063794 for 12 hours (lane 2), 24 hours (lanes 3), 48 hours (lane 4) or 72 hours (lane 5). Proteins were visualised by Western blotting using the antiserum indicated. **D**. MGMT protein levels in **(C)** were quantified and expressed relative to the α tubulin loading control. Error bars are the SE (n = 3). Confidence limits were set: *p = <0.2, **p = <0.05 ***p = <0.005. **E**. Cells were incubated in the absence or presence of KU0063794 as indicated. Mitochondrial activity (a measure of cell viability) was assessed using an MTS assay as described in Materials and Methods and is expressed relative to untreated cells (set at 100%). Error bars are the S.D (n = 3).

Mutations in mTOR signalling pathways in GBM cells can result in hyperactive PI3-K/mTOR signalling, promoting cell survival, protein synthesis and cell proliferation [9,10,20]. KU0063794 directly inhibits this pathway and should therefore prevent the cells from proliferating and reduce their ability to synthesise new protein. To this end, cells were treated with KU0063794 for 72 hours and protein levels monitored by SDS-PAGE and immunoblotting. As expected, phosphorylation of 4E-BP1 on either Ser65 or Thr70, and rpS6 on Ser240/244 were abrogated within 12 hours of treatment of the cells with KU0063794 (Figure 3A lane 2 vs. lane 1), which indicates reduced translation initiation. At early time points, KU0063794 had little effect on the phosphorylation of ERK1/2, p38MAPK or eIF2α (Figure 3A, lanes 1–3), but phosphorylation was decreased at later time points (lanes 4 and 5). There was no change in the total level of these proteins during the incubation period (data not shown). In contrast, the phosphorylation of Akt on Thr308 (an indicator of Akt activity) increased with time (lanes 2–5 vs. lane 1) reflecting a previously reported feedback activation of PI3K and PDK1 following mTORC1 inhibition [13,14].

mTORC1, when active, regulates protein translation through the phosphorylation of 4EBP1. Phosphorylation of 4E-BP1 prevents it binding to eIF4E, which enables eIF4E to participate in the formation of the eIF4F complex on the m^7^ GTP cap structure of mRNA and mediate small ribosomal subunit binding and subsequent protein translation [11]. As described above, cells were incubated for 72 hours and the binding of eIF4E and associated proteins with the 5’ end of mRNA was measured using Sepharose beads coupled with m^7^ GTP. Bound protein was recovered and visualised using SDS-PAGE and Western blotting alongside eIF4E and associated proteins isolated from untreated cells. As expected, inhibition of mTORC1/2 signalling pathway resulted in an increase in the binding of hypophosphorylated 4E-BP1 to recovered eIF4E and a moderate decrease in eIF4G associated with eIF4E (Figure 3B). Unsurprisingly this observation was coupled with a reduction in protein synthesis rates by around 70% upon treatment of KU0063794 for 48 hours (Figure 2C) and at 72 hours, the KU0063794 treated cells were synthesising protein at less than 10% of the rate of the untreated control.The levels of MGMT protein were also monitored in cells treated with KU0063794, as this information was necessary to identify if the rise in MGMT levels during the combined treatment of KU0063794 and TMZ was solely caused by KU0063794 or if the effect observed was synergistic. MGMT protein levels were not only maintained during this treatment (Figure 3C, lanes 2–5 vs. lane 1), but in a similar fashion to cells treated with both KU0063794 and TMZ, protein levels increased over a 72 hour period, despite clear inhibition of mTORC1 (as shown by dephosphorylation of 4E-BP1, Figure 3C). Quantification of these data from three separate experiments show that, relative to the alpha tubulin protein loading control that MGMT protein levels were increased by statistically significant levels at 48 and 72 hours (p = 0.08 and 0.2, respectively) in cells treated with KU0063794 alone compared with untreated controls (Figure 3D).

This observation clearly shows that the rise in MGMT protein is caused by KU0063794. Yet it was not clear if the rise in MGMT protein was protein specific or if mTORC1/2 inhibition brought about a general inhibition of protein degradation. To investigate this, we analysed the steady state level of p21^cip1^, which is known to have a short half-life (approximately 100 minutes [21]). The disappearance of p21^cip1^ protein after 12 hours indicates that KU0063794 treatment does not cause a general inhibition of protein degradation (Figure 3C), suggesting that the increase in MGMT protein levels was indeed specific.

Cell viability (Figure 3E) and cell cycle progression in T98G cells (Additional file 1: Figure S6) was also investigated. KU0063794 had little effect on metabolic activity (Figure 3E) but did cause a reduction in the S phase population and a small increase in the sub-G1 population (Additional file 1: Figure S6), indicative of an inhibition of the cell cycle and some apoptosis at later incubation times.

To ensure that the effects observed during treatment of all of the compounds were not cell specific, we also used U87-MG cells. This glioma cell line expresses very low levels of MGMT, which is confirmed by the absence of MGMT protein when visualised by Western blotting (Additional file 1: Figures S7, S8 and Additional file 2: Figure S9). Both KU0063794 and TMZ generally had a similar effect on the U87-MG cell line compared to the T98G cell line. However, there were some differences in responses observed here. The most obvious differences were a less robust cessation of cell cycle progression in U87-MG cells in response to KU0063794 and subtle differences on mTORC1/2 and eIF2 alpha signalling pathways. In U87-MG cells incubated with TMZ, there was no change in 4E-BP1 or T308 phosphorylation but there was an increase in eIF2 alpha phosphorylation, suggesting that inhibition of translation under these conditions in U87-MG cells may have been caused by eIF2 alpha phosphorylation.

### mTOR1/2 inhibition results in selective translation of MGMT during reduced protein synthesis rates

**Figure 4:**
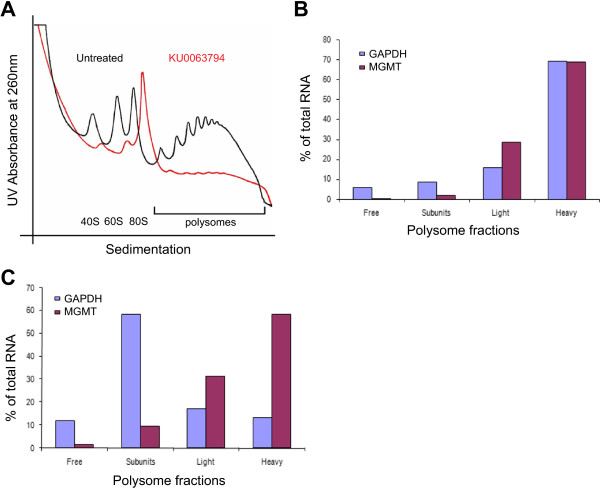
**KU0063794 inhibits global protein translation**, **while increasing translation of the MGMT protein.** Cells were incubated in the absence or presence of KU0063794. **A**. Cell lysates were prepared, polysomes fractionated using sucrose density gradients. The UV profile from this analysis is shown here and the fractionation of 40S, 560S, 80S ribosomes and polysomes is indicated. mRNA from pooled fractions was subsequently extracted as described in the Materials and Methods. For each gradient, qRT-PCR was used to determine levels of GAPDH and MGMT mRNA distribution across each fraction (free represents RNA and associated material at the top of the gradient and subunits show 40S/60S/80S-bound RNA), with the total mRNA in each gradient set at 100%. Cells were incubated for 24 hours in the absence **(B)** or presence of KU0063794 **(C)**.

Considering that a combination of TMZ and KU0063794 did not, as expected, deplete the levels of MGMT protein (although KU006374 treatment clearly causes a stark dephosphorylation of 4E-BP1 and a massive inhibition of eIF4F complex formation), we hypothesised that the MGMT mRNA may be post-transcriptionally regulated. To investigate this further, polysome gradient analysis was performed on cell extracts to monitor specific mRNAs associated with ribosomes and hence which are undergoing active translation. As predicted, polysomes were disaggregated in the presence of KU0063794, indicative of an inhibition of protein synthesis (Figure 4A). Using qRT-PCR, we specifically investigated the translational profile of MGMT mRNA across fractions from the sucrose density gradients. In the absence of drug treatment both control mRNA, GAPDH, and MGMT mRNA are preferentially localised in the polysomes, especially the heavy polysomes, and therefore reflect a high degree of translation (Figure 4B). Treatment of cells with KU0063794 for 24 hours promoted the relocation of a control mRNA, GAPDH, from heavy polysomes to 80S monosomes (Figure 4C). This finding is consistent with the observed inhibition of protein synthesis (Figure 2C), as during reduced levels of protein synthesis cap-dependant translation is generally inhibited, resulting in a reduced amount of polysomes associated with mRNA. Surprisingly, although global protein synthesis rates were reduced, MGMT mRNA remained associated with heavy polysomes when cells were incubated for 24 hours with KU0063794 (Figure 4C). This could reflect maintenance of translation of MGMT mRNA or else protection of this mRNA in stalled polysomes; further work would be required to elucidate this phenomenon.

We also confirmed that total mRNA expression did not change on KU0063794 treatment using qRT-PCR (Additional file 2: Figure S10). MGMT mRNA levels remained unchanged at 24 hours compared to untreated cells under all incubation conditions tested (relative to 18S rRNA).

### Inhibition of mTOR signalling does not affect the half-life of MGMT protein in T98G cells but stabilises MGMT protein in the presence of TMZ

**Figure 5:**
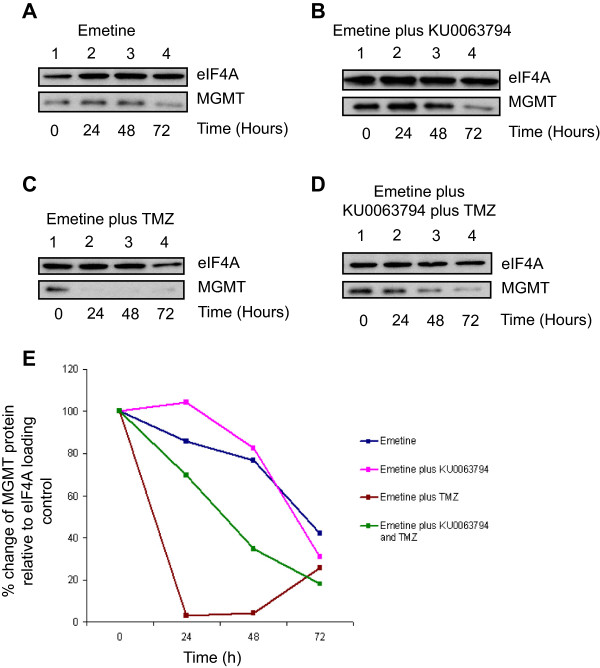
**Inhibition of mTOR signalling does not affect the half-life of MGMT protein in T98G cells but stabilises MGMT in the presence of TMZ.** T98G cells were incubated in the presence of emetine alone **(A)** or emetine and KU0063794 **(B)**, emetine and TMZ **(C)** or emetine, KU0063794 and TMZ **(D)** for the times indicated. Western blotting was carried out as described in Materials and Methods and the antiserum used indicated. **E**. Western blots from the representative data shown were used to quantify MGMT protein levels using eIF4A as a loading control, as described above. MGMT protein levels are expressed relative to those found in untreated cells (set at 100%).

As inhibition of mTOR kinase activity is seen to inhibit global protein synthesis rates yet increase the steady state level of MGMT protein without affecting total mRNA levels, we investigated whether this reflects a change in the half-life of MGMT protein. To this end, cells were incubated in the presence of emetine (alone) at a level to block protein synthesis totally (Figure 5A, data not shown), or a combination of emetine and KU0063794 (Figure 5B), TMZ (Figure 5C), or KU0063794 and TMZ together (Figure 5D,E). MGMT protein was relatively stable in T98G cells, with a half-life of approximately 60 hours (Figure 5A). This did not change when cells were incubated in the presence of KU0063794 (Figure 5B). However, the addition of TMZ reduced the half-life of MGMT protein in T98G cells to less than 12 hours. This reflects the fact that MGMT undergoes proteolysis after undergoing methylation as part of its DNA repair function [6]; as emetine is entirely blocking protein synthesis, MGMT is unable to be re-synthesised to maintain steady state levels (Figure 5C). Inhibition of mTOR signalling with KU0063794 concomitant with TMZ partially restored steady state levels of MGMT (Figure 5D). These data (quantified in Panel E from the representative Western blots) suggest either that reduced mTOR activity promotes MGMT stability or that KU0063794 interferes with the proteasomal destruction of MGMT post-repair.

## Discussion

GBM is the most aggressive adult brain cancer and TMZ, an oral methylating chemotherapeutic agent, is part of the standard treatment, often accompanied by radiotherapy [2-4]. TMZ exerts its antitumour effect by methylating guanine at a number of sites [22]. The N^7^ guanine and N^3^ adenine adducts caused by TMZ are repaired by the base excision repair pathway, but the O^6^-methyl-guanine (O^6^MeG) lesion cannot be repaired in this fashion, making it the most potently cytotoxic adduct [22]. O^6^MeG pairs with thymine and leads to GC-AT transitions, beginning a cycle of futile DNA mismatch repair which causes DNA double-stranded breaks and, ultimately causes tumour cell death [6,22,23].

MGMT is a DNA repair protein that repairs the O^6^MeG lesion created by TMZ by a process of irreversible binding and subsequent degradation of the protein [5,23]. When O^6^MeG is repaired by MGMT, the majority of cells become resistant to O^6^MeG-triggered apoptosis [4-6]. Hence the level of MGMT activity correlates with resistance of tumour cells to TMZ [24]. As mutations in the mTOR signalling pathway in GBM frequently result in hyperactive PI3-K/mTOR signalling, promoting cell survival and protein synthesis [9,10,20], we investigated whether mTOR kinase activity has a role in regulating levels of MGMT protein expression in human glioblastoma cells.

As predicted, we show that TMZ does not reduce MGMT protein levels in T98G cells, so the large amount of MGMT in the cells is effectively repairing any lesions created by TMZ. This suggests that, in agreement with previous studies [25-27], on its own TMZ is not sufficiently effective in cellular environments containing MGMT overexpression.

In contrast to cells treated with TMZ alone, when protein loading was accounted for in three separate experiments, our data show that in cells treated with both KU0063794 and TMZ, there was an increase in MGMT protein expression. This occurred even though global protein synthesis rates were reduced by around 80% under these conditions. These data suggest that although dual mTOR inhibition was indeed impairing the formation of the translation initiation complex, reducing global protein synthesis rates and causing a disaggregation of polysomes from mRNA, protein levels were still increasing. To decipher if this unexpected maintenance of MGMT protein levels was reliant on an alternative translation mechanism, we assessed the translation status of MGMT mRNA. This demonstrated that during conditions of reduced global translation rates, and therefore reduced mRNA association with polysomes, MGMT mRNA remained associated with heavy polysomes, although GAPDH mRNA shifted to sub polysomal fractions. This is strong evidence that MGMT mRNA might be translated using a cap-independent mechanism, or preferentially recruited to ribosomes under conditions of low levels of eIF4F [28], but in the absence of increased levels of eIF2α phosphorylation (Figure 3). This is further supported by the presence of a number of GC-rich short stem loops in the annotated 3’-UTR of the *MGMT* mRNA, which may also have a role in this process. Additional extensive biochemical analysis would be needed to determine if this is the case. Alternative mechanisms controlling translation have been identified under hypoxic stress conditions, which rely on protein complexes involving the alternative eIF4E isoform eIF4E2, specifically recruiting target mRNAs to polysomes during periods of reduced cap dependant translation [29]. These findings could compliment the preferential translation of MGMT under stress conditions, but further investigations would need to be carried out to confirm this.In addition to the mechanisms regulating translational control of MGMT mRNA, the stability of the MGMT protein was also investigated, as this is of paramount importance when considering its manipulation for effective chemotherapeutic treatment. The inhibition of protein synthesis elongation with emetine in the presence of TMZ caused a dramatic decrease in MGMT protein level, reflecting the rapid degradation of the protein following DNA repair activity (Figure 5). However, the combination of KU0063794 and TMZ compared with TMZ alone resulted in a lengthened half-life of MGMT protein to around 40 hours. This is an important observation, as an increase in MGMT stability has the potential to greatly impede treatment of GBM with DNA methylating agents.

Previous work has used the mTORC1 inhibitor rapamycin to inhibit the mTOR/PI3K pathway in an attempt to inhibit the growth of GBM [30-32]. It was therefore a logical progression to investigate specific mTOR signalling inhibition in GBM cells and their possible use in conjunction with TMZ. Even though research in this area is surprisingly limited, recent studies have indicated combination treatments involving compounds that negatively regulate the mTOR signalling pathway may impact MGMT regulation [33], further compounding the need for research to increase the efficacy of TMZ. Indeed a number of clinical trials combining mTOR inhibitors with radiation and TMZ have been initiated and are recruiting patients (e.g. everolimus (RTOG 0913) and BKM120).

The data presented here reveal that *in vitro*, rather than inhibiting translation of MGMT, mTOR inhibition promotes steady state levels of the MGMT protein and counteracts the depleting effects of TMZ. We therefore conclude that further *in vivo* analysis of mTOR inhibition is necessary to determine if this effect is also found in patients exhibiting GBM. If so, mTOR inhibition would not be a useful adjunct to TMZ therapy in a clinical setting and could exacerbate tumour growth.

## Competing interests

The author’s declare that they have no competing interests.

## Authors’ contributions

Sarah Smalley, Simon Morley and Anthony Chalmers designed the research. Sarah Smalley carried out all experiments and drafted the paper. All authors read and approved the final manuscript.

## Supplementary Material

Additional file 1: Figures S1 & S2 The effect of TMZ on cell cycle progression in T98G cells. S1. T98G cells were incubated with drugs as indicated for 24 hours and then harvested for FACS analysis. Error is S.D (n = 3). S2. T98G cells were incubated as in S1. An MTS assay was performed. Results are expressed relative to untreated cells. Error is S.D (n = 3). **Figure S3.** TMZ does not affect mTORC1 signalling in T98G cells. T98G cells were incubated as in S1 and proteins visualised by WB. **Figure S4.** TMZ does not affect the inhibition of mTORC1 signalling by KU0063794 in T98G cells. T98G cells were incubated with drugs as indicated and proteins visualised by WB. **Figure S5.** TMZ does not alter the distribution of MGMT in T98G cells. T98G cells were incubated with drugs as indicated. Cells were processed for IF visualisation of MGMT. The localisation of MGMT was quantified in the nuclear and cytoplasmic regions. **Figure S6.** The effect of KU0063794 on cell cycle progression in T98G cells. T98G cells were incubated as in S4 and harvested for FACS analysis. Error is S.D (n = 3). **Figure S7.** The effect of KU0063794 and TMZ on cell cycle progression in U87-MG cells. U87-MG cells were incubated as in S5 and harvested for FACS analysis. Error is S.D (n = 3). **Figure S8 and S9.** KU0063794 inhibits mTOR signalling and protein synthesis in U87-MG cells. S8. U87-MG cells were incubated as in S7 and proteins visualised by WB. S9. Incorporation of radioactive methionine into cellular protein was measured. Error is S.D (n = 3). **Figure S10.** The effect of KU0063794 on mRNA levels. T98G cells were incubated as in S5 for 24 hours. Levels of MGMT mRNA is expressed as a fold change, relative to levels in untreated cells. Click here for file

Additional file 2: Figure S9. One hour prior to harvesting at 24 hours, cells were incubated with [^35^S] methionine, as described in Materials and Methods. Extracts were prepared and incorporation of radioactive methionine into protein determined as cpm/μg protein; results are presented as a % of the rate obtained in cells incubated in the absence of TMZ incubated for 72 hours. Error bars are the S.D (n = 3). **Figure S10.***The effect of KU0063794 on mRNA levels*. T98G cells were incubated in the absence or presence of 10 μM KU0063794, 10 μM TMZ or both 10 μM KU0063794 and 10 μM TMZ for 24 hours and mRNA was extracted from cell extracts as described in Materials and Methods. For each treatment, quantitative RT-PCR was used to determine levels of MGMT mRNA relative to 18rRNA, as described. Data are presented as a fold change in amount relative to levels observed in untreated cells. Error bars are the S.D (n = 3). Click here for file
