# Supplementary figures and images for: mTOR inhibition and levels of the DNA repair protein MGMT in T98G glioblastoma cells
Source: Mol Cancer. 2014 Jun 8;13:144. doi: 10.1186/1476-4598-13-144 (PMC4061125; doi:10.1186/1476-4598-13-144)

Supplementary figures

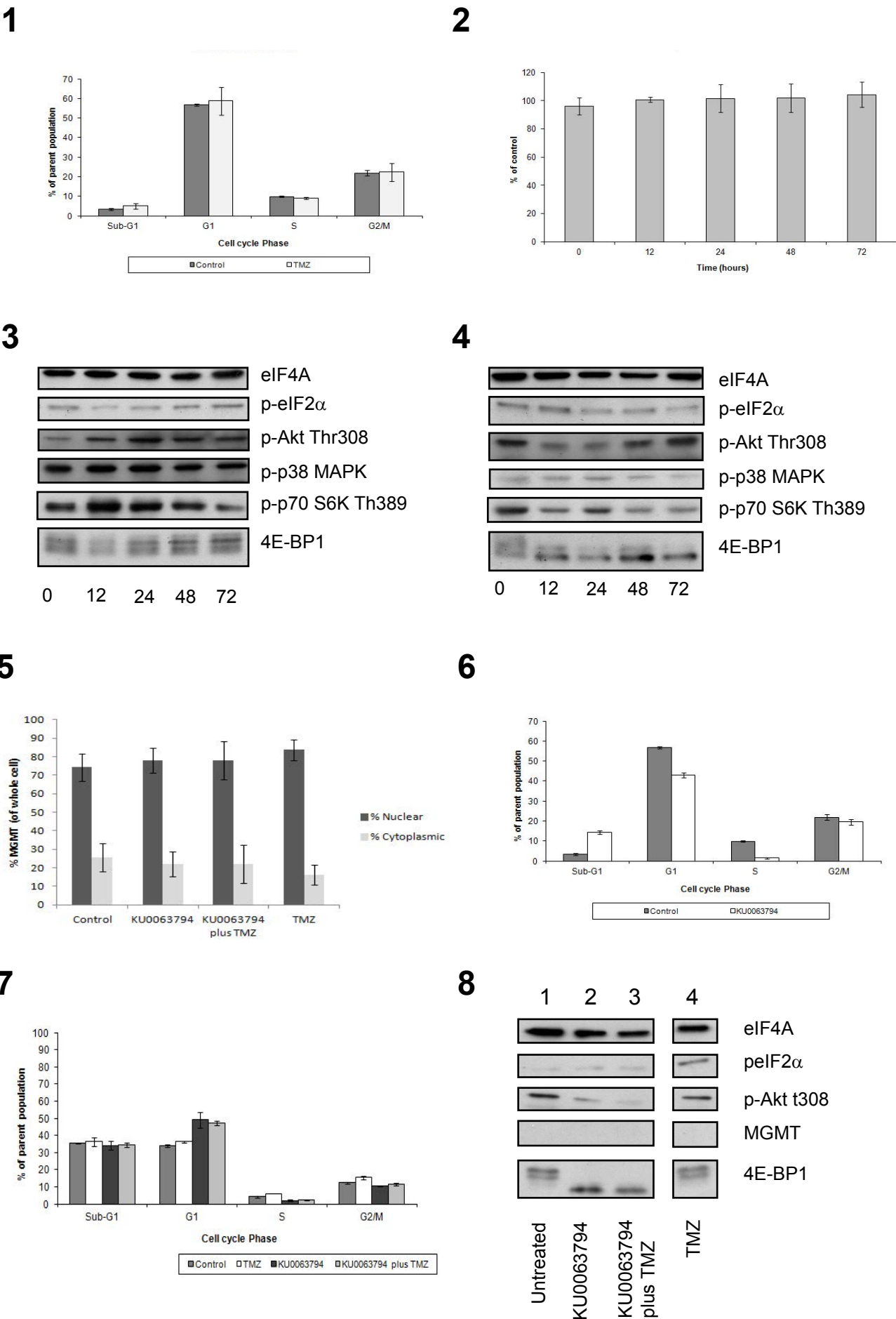

Supplement: Additional file 1: Figures S1 & S2 — The effect of TMZ on cell cycle progression in T98G cells. S1. T98G cells were incubated with drugs as indicated for 24 hours and then harvested for FACS analysis. Error is S.D (n = 3). S2. T98G cells were incubated as in S1. An MTS assay was performed. Results are expressed relative to untreated cells. Error is S.D (n = 3). Figure S3. TMZ does not affect mTORC1 signalling in T98G cells. T98G cells were incubated as in S1 and proteins visualised by WB. Figure S4. TMZ does not affect the inhibition of mTORC1 signalling by KU0063794 in T98G cells. T98G cells were incubated with drugs as indicated and proteins visualised by WB. Figure S5. TMZ does not alter the distribution of MGMT in T98G cells. T98G cells were incubated with drugs as indicated. Cells were processed for IF visualisation of MGMT. The localisation of MGMT was quantified in the nuclear and cytoplasmic regions. Figure S6. The effect of KU0063794 on cell cycle progression in T98G cells. T98G cells were incubated as in S4 and harvested for FACS analysis. Error is S.D (n = 3). Figure S7. The effect of KU0063794 and TMZ on cell cycle progression in U87-MG cells. U87-MG cells were incubated as in S5 and harvested for FACS analysis. Error is S.D (n = 3). Figure S8 and S9. KU0063794 inhibits mTOR signalling and protein synthesis in U87-MG cells. S8. U87-MG cells were incubated as in S7 and proteins visualised by WB. S9. Incorporation of radioactive methionine into cellular protein was measured. Error is S.D (n = 3). Figure S10. The effect of KU0063794 on mRNA levels. T98G cells were incubated as in S5 for 24 hours. Levels of MGMT mRNA is expressed as a fold change, relative to levels in untreated cells. [file 1476-4598-13-144-S1.pdf]

9

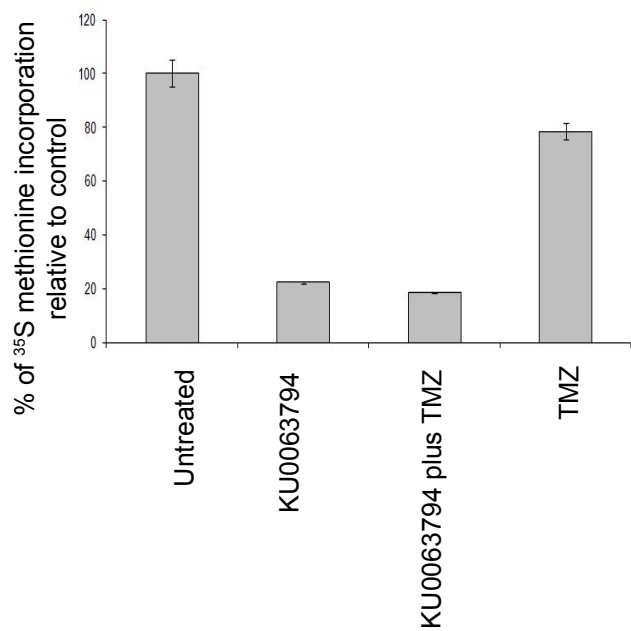

10

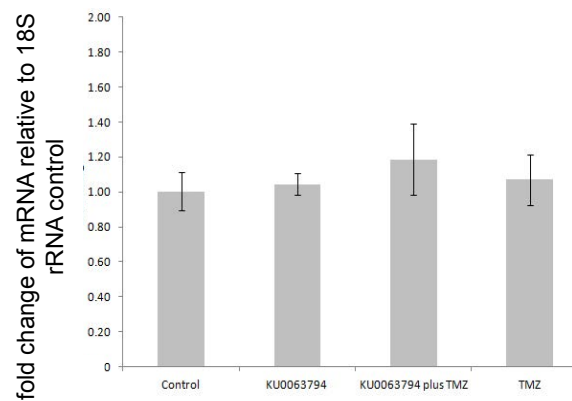

Supplement: Additional file 2: Figure S9. — One hour prior to harvesting at 24 hours, cells were incubated with [35S] methionine, as described in Materials and Methods. Extracts were prepared and incorporation of radioactive methionine into protein determined as cpm/μg protein; results are presented as a % of the rate obtained in cells incubated in the absence of TMZ incubated for 72 hours. Error bars are the S.D (n = 3). Figure S10.The effect of KU0063794 on mRNA levels. T98G cells were incubated in the absence or presence of 10 μM KU0063794, 10 μM TMZ or both 10 μM KU0063794 and 10 μM TMZ for 24 hours and mRNA was extracted from cell extracts as described in Materials and Methods. For each treatment, quantitative RT-PCR was used to determine levels of MGMT mRNA relative to 18rRNA, as described. Data are presented as a fold change in amount relative to levels observed in untreated cells. Error bars are the S.D (n = 3). [file 1476-4598-13-144-S2.pdf]
